# Formulation and Evaluation of Chitosan-Gelatin Thermosensitive Hydrogels Containing 5FU-Alginate Nanoparticles for Skin Delivery

**DOI:** 10.3390/gels8090537

**Published:** 2022-08-26

**Authors:** Asif Nawaz, Shafi Ullah, Maha Abdallah Alnuwaiser, Fazal Ur Rehman, Samy Selim, Soad K. Al Jaouni, Arshad Farid

**Affiliations:** 1 Advanced Drug Delivery Lab, Gomal Centre of Pharmaceutical Sciences, Faculty of Pharmacy, Gomal University, Dera Ismail Khan 29050, Pakistan; 2 Department of Chemistry, College of Science, Princess Nourah bint Abdulrahman University, P.O. Box 84428, Riyadh 11671, Saudi Arabia; 3 Department of Clinical Laboratory Sciences, College of Applied Medical Sciences, Jouf University, Sakaka 72388, Saudi Arabia; 4 Department of Hematology/Oncology, Yousef Abdulatif Jameel Scientific Chair of Prophetic Medicine Application, Faculty of Medicine, King Abdulaziz University, Jeddah 21589, Saudi Arabia; 5 Gomal Center of Biochemistry and Biotechnology, Gomal Univesity, Dera Ismail Khan 29050, Pakistan

**Keywords:** thermosensitive hydrogels, nanoparticles, 5FU, chitosan, alginate, skin delivery

## Abstract

(1) Background: Chitosan-gelatin-based thermosensitive hydrogel containing 5FU-alginate nanoparticles was formulated for the effective and sustained delivery of 5FU to the skin. (2) Methods: Alginate, a polysaccharide was used for the formulation of nanoparticles using a spray drying technique. Size, zeta potential, and surface morphology were investigated using a zetasizer and scanning electron microscope. The hydrogel was fabricated using chitosan and gelatin. Several important analyses were used to characterize these prepared topical hydrogels. The pH, visual transparency, rheological behavior, and swelling index of the prepared hydrogels were evaluated. The in vitro release studies were performed at different pH (5.5 and 7.4) and temperature (32 and 37 °C) conditions using a Franz diffusion cell. Ex vivo permeation and in vivo studies were performed using Sprague Dawley rats. (3) Results: Results show that spherical nanoparticles were produced at sizes of 202–254 nm and with zeta potentials of −43 to −38 mV. The prepared nanoparticles were successfully incorporated into chitosan-gelatin-based hydrogels using a glycerol 2-phosphate disodium salt hydrates crosslinker. Drug polymers and excipients compatibility and formulation of hydrogels was confirmed by ATR-FTIR results. The pH of the prepared hydrogels was in accordance with the skin pH. The viscosity of prepared hydrogel increased with temperature increase and phase transition (sol-gel transition) occurred at 34 °C. The release of drug was sustained in case of nanoparticles incorporated hydrogels (5FU-Alg-Np-HG) as compared to nanoparticles (5FU-Alg-Np) and simple hydrogels (5FU-HG) (ANOVA; *p* < 0.05). The premature and initial burst release of 5FU was prevented using 5FU-Alg-Np-HG. The release mechanism of 5FU from the 5FU-Alg-Np-HG diffusion was followed by swelling and erosion, as suggested by Korsmeyer-Peppas model. The prepared hydrogel proved to be non-irritant. Ex vivo permeation study across rat’s skin suggests that permeability of nanoparticles (5FU-Alg-Np) was higher than the 5FU-Alg-Np-HG (ANOVA; *p* < 0.05). However, skin-related drug retention of 5FU-Alg-Np-HG was significantly higher than the 5FU solution, 5FU-Alg-Np, and 5FU-HG (ANOVA; *p* < 0.05). This was due to swelling of hydrogels in the lower layers of skin where the temperature is 37 °C. The higher concentration of 5FU in the skin is helpful for treatment of local skin cancer, such as melanoma, and actinic keratosis. In vivo results also confirmed maximum AUC, t_1/2_, and skin-related drug retention of 5FU-Alg-Np-HG. (4) Conclusions: Chitosan-gelatin-based hydrogels containing 5FU-Alg-Np possess exceptional properties, and can be used for the sustained delivery of 5FU for the treatment of local skin cancers.

## 1. Introduction

Hydrogels is one of the most important biomaterials. Hydrogels possess a highly porous 3D arrangement of interconnected and crosslinked polymers containing a high portion of aqueous phases or biological fluids (higher than 20%), due to which, its network is not dissolved through chemical and physical interactions [1,2,3,4]. Hydrogel possess very good biocompatibility and capability of embedding hydrophilic agents in therapeutic area owing to its high water contents [5]. Hydrogels have great flexibility in terms of composition and ability to adjust to various administration routes, ranging from parenteral (such as intramuscular (i/m), intradermal, etc.) to non-parenteral (such as topical, oral, etc.) [6]. Currently, hydrogels are an attractive type of site-specific drug delivery system, and have many applications in medical and biomedical engineering, including wound and cartilage regeneration, bone tissue engineering, soft robotic components, biosensors, and inflammation relief [7,8]. Hydrogels are used for preparation/manufacturing of contact lenses, scaffolds, wound dressing, and hygiene products [9,10]. The unique properties of hydrogels, such as high water content, flexibility, softness, and biocompatibility, makes them suitable for topical/transdermal delivery.

Recently, stimuli-responsive hydrogels are gaining great attention due to their ability to control the release of drugs and bioactive molecules. These types of hydrogels respond to several stimuli to change their physical, chemical, and/or mechanical properties to favor drug release at the target site [11]. The development of smart biomaterials has recently been stimulated by the increased focus on precision medicine and individualized medication [12]. As “smart biomaterials”, stimuli-responsive hydrogels can release drugs in response to external triggers such as temperature, pH, magnetic and electrical fields, biomolecule concentration, and light [13]. In reaction to a tiny external trigger, smart hydrogels abruptly modify both their macroscopic and physical properties. This hydrogel distinctiveness stems from their non-linear feedback [14,15]. In fact, they can change their phase volume in response to triggers in a way that is intensity-scalable, reversible, repeatable, and predictable, and they can do so after the trigger has been removed [16]. Such transitions include alterations in the solvent interactions, physical state, shape, conductivity, solubility, and hydrophilicity [17]. Two significant characteristics that are frequently studied in depth are pH and temperature. This is so that the hydrogels can perform as intended in the target location at body temperature and pH levels that are physiological [18]. High-molecular-weight polymers known as pH-responsive hydrogels (PRHs) vary in volume or phase in response to changes in the pH of an external medium [19]. The pH of the surrounding environment at the pKa and pKb values of the pendant basic and acidic groups influences the swelling of PRHs [20]. Variations in the pH can occur inside the body due to certain disorders such as inflammation, chronic wounds, and tumors, which are targeted by the drug delivery system [9].

Nanoparticles (NPs) made from biodegradable natural polysaccharides are of great interest for the delivery of peptide, protein, and other drugs that require controllable release behavior [21]. The naturally occurring polysaccharides, which are widely used to obtain controlled drug release behavior, include alginate, chitosan, cyclodextrin (CD), and cellulose [22]. Alginate and chitosan are particularly promising as drug delivery vehicles due to their low toxic effects [23], biodegradability, and biocompatibility. Both the polymers are widely used in drug delivery as rate-controlling polymers, and used in the formulation of nanoparticles, microparticles, scaffolds, and hydrogels. Chitosan is a cationic polymer, widely employed as rate-controlling polymer and permeation enhancer in topical/transdermal delivery [24]. Gelatin has been extensively used as a biomaterial for soft and hard tissue engineering and drug delivery due to its excellent biocompatibility, biodegradability, and non-immunogenicity properties [25]. Gelatin is one of the most widely used polymer in hydrogel formation. Gelatin hydrogels are formed by physical crosslinking in water at concentration of 2% *w*/*v* and temperature 30–34 °C. Gelatin can form a high-molecular-weight network in the presence of a crosslinker that is capable of swelling [26].

The chemotherapeutic agent,5-fluorouracil (5FU), is most frequently used in the treatment of cancer. However, its short biological half-life (5–10 min) is the main problem that limits its application. This is due to its fast metabolism by liver enzyme dihydropyrimidine dehydrogenase [27]. Additionally, 5FU has a number of toxic side effects that are noticeable in the gastrointestinal system and bone marrow, as well as non-selective bioactivity against healthy normal cells. A viable substitute for minimizing these side effects and maximizing drug targeting and therapeutic advantages is topical administration of 5FU [28].

To improve the clinical efficiency of 5-fluorouracil, 5FU-alginatenanoparticles were prepared and incorporated into chitosan-gelatin hydrogels. Previously, different formulation techniques were used for local skin delivery, such as nanoemulsions, microemulsions, solid lipid nanoparticles, nanostructured lipid carriers, liposomes, and nanoparticles [29,30]. The main problem associated with the local skin delivery system is the retention time of the drug in the skin. Therefore, to increase the retention time of drugs in the skin, thermosensitive hydrogels were developed. Until now, no study has been carried out on the formulation of nanoparticle-loaded chitosan-gelatin thermosensitive hydrogels for the effective local delivery of 5FUto the skin. The aim of development of this system was to control the premature release of 5FU and maximize the skin-related drug retention in order to target the local skin cancer, for example, melanoma.

## 2. Results and Discussion

### 2.1. Preparation and Characterization of Nanoparticles

The Alg-5FU nanoparticles were prepared using a spray drying technique;5FU can be encapsulated in the matrix of the polysaccharides via bonds with functional groups in the alginate chain [31]. Particle size and zeta potential are the principal criteria for evaluating effective anticancer drug delivery targeting tumor tissue [32]. Studies have shown that nanoparticles in the size range of 20–200 nm have the maximum retention in the tumor cell [33].The particle size of the prepared nanoparticles was determined in an aqueous solution. It was found that the particle size of nanoparticles is in the range 202–254 nm. Nanoparticles 50–500 nm in size are acceptable for transdermal drug delivery [34]. The formulated nanoparticles were of sizes within acceptable range, and thus suitable for skin delivery. The particle size of nanoparticles slightly increases with the addition of 5FU (*t*-test; *p* < 0.05), confirming the encapsulation of 5FU into the matrix of crosslinks formed by alginate chains in nanoparticles.

**Figure 1:**
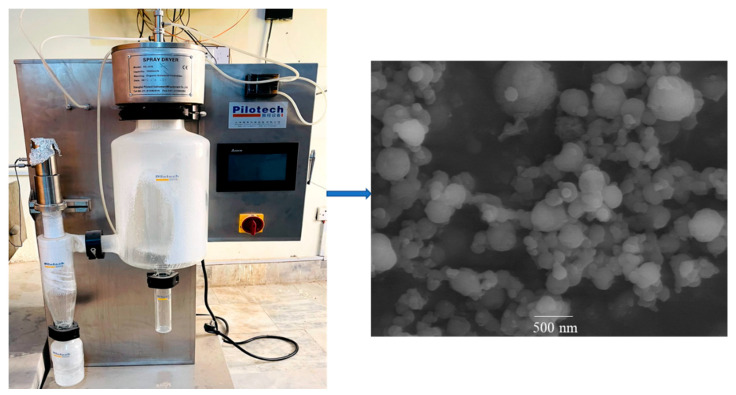
Photograph of spray dryer and SEM of 5FU-Alg-Np.

Table 1 showsthe zeta potential results of the prepared nanoparticles. The prepared nanoparticles had negative zeta potential, because of the negative zeta potential of the alginate component [32]. The blank nanoparticle had a high surface charge of −43.67 mV while the presence of 5FU slightly increased the surface charge (−38.72 mV). The highly negative zeta potential induced repulsion between nanoparticles, greatly reducing the propensity to aggregate and making the nanoparticles highly stable in an aqueous solution [35]. The zeta potential values greater than +30 or less than −30 mV indicate the stability of nanoparticles, and helps to prevent nanoparticles aggregation [36]. Thus, the alginate-based nanoparticles protect the drug, and is an ideal carrier for a drug delivery system. The SEM images (Figure 1) show that the nanoparticles possess a spherical shape with a smooth surface.

### 2.2. Preparation and Characterization of Hydrogels

The 5FU solution and 5FU-Alg-Np-loaded chitosan-gelatin-based hydrogels were prepared. Chitosan and gelatin solutions when mixed together, electrostatic interaction occurs, which results in interpenetrating self-assemble soft aqueous pores as a result of amide bonds formation [37]. These soft pores were filled with 5FU solution and/or 5FU-Alg-Np to form drug-loaded hydrogels.

#### 2.2.1. ATR-FTIR Analysis

**Figure 2:**
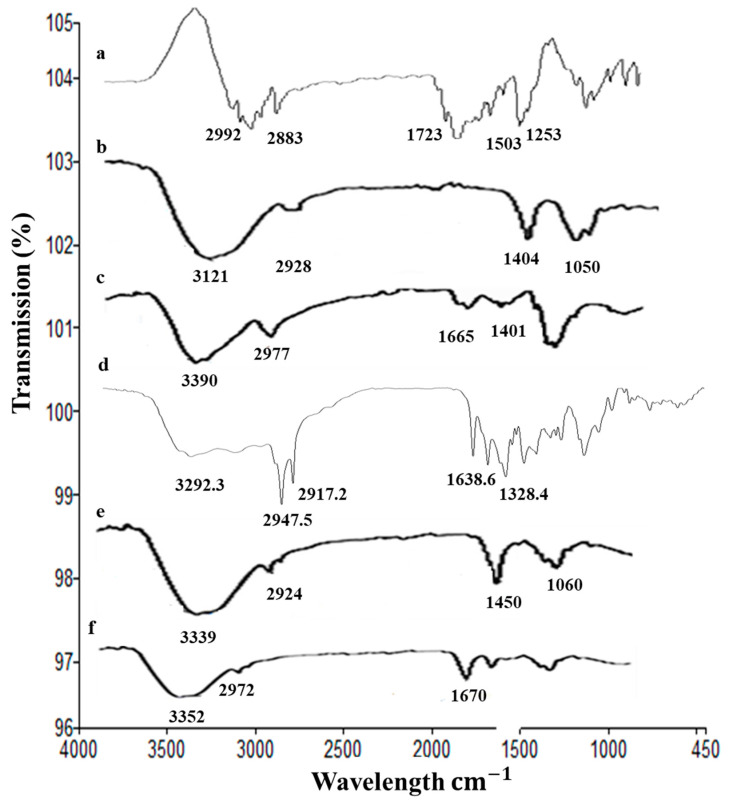
ATR-FTIR spectra of (**a**) 5FU, (**b**) alginate, (**c**) chitosan, (**d**) gelatin, (**e**) 5FU-Alg-Np, and (**f**) 5FU-Alg-Np-HG.

The ATR-FTIR spectra of drug 5FU, alginate, chitosan, nanoparticles, and hydrogels are shown in Figure 2. We found that 5FU has the characteristic peaks at 2992 (N–H stretching vibrations), 2883 (C=H stretching vibrations), 1723 (C=O group of ketone), 1505 and 1253 cm^−1^ (stretching vibration of C=N and C=F, respectively) [38]. The characteristic FTIR peaks of chitosan were at 3390, 2977, 1665, and 1401 cm^−1^, and for alginate were at 3121, 2928, 1404, and 1050 cm^−1^. The characteristics peaks of gelatin were observed at 3292 cm^−1^ (N–H stretching), 2947 cm^−1^ (C–H stretching), and 1637 cm^−1^ (C=O stretching). The FTIR spectrum of nanoparticles have the characteristic absorption peaks at 3339, 2924, 1450, and 1060 cm^−1^, showing the absorption bands shift to new positions as compared 5FU. This indicates the successful encapsulation of 5FU into alginate nanoparticles. The absorption peaks of nanoparticles and 5FU were weaken in FTIR spectrum of hydrogels (Figure 2). This confirms the incorporation of nanoparticles into the chitosan-based hydrogels.

#### 2.2.2. pH and Visual Transparency of Hydrogels

**Figure 3:**
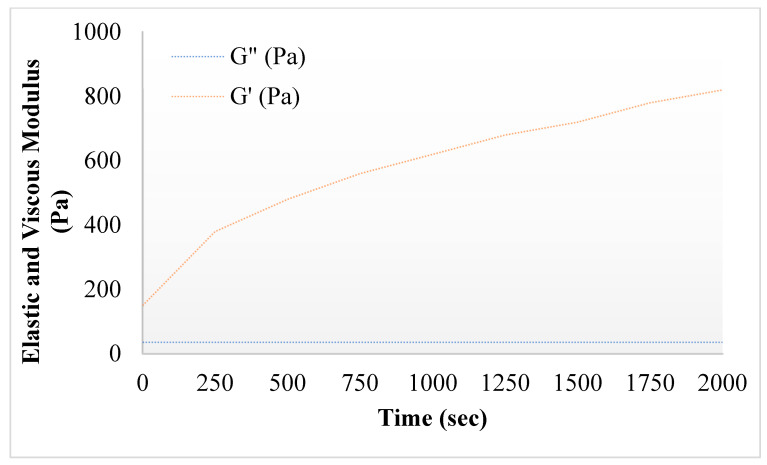
Elastic and viscous modulus of 5FU-Alg-Np-HG.

The pH is an important parameter for topical formulations. The pH of all the prepared hydrogels was checked using digital pH meter (Table 2). The pH of the hydrogel (6.83) was slightly higher than its sol form (6.27). The results show that there is no drastic change in pH value after conversion of sol to gel, confirming that there is no undesired chemical reaction in the system. This pH range is also in correspondence to biological skin pH, hence no irritation on skin surface will be caused. Skin pH ranges from 4 to 6, and the topical formulations should possess pH in this range [39]. Visual evaluation of the prepared hydrogel was performed at different temperatures (i.e., 25−37 °C) for the assessment of transparency. The chitosan-gelatin hydrogel was transparent at 25 °C, which proves that all the added ingredients are homogeneously mixed. It was observed that the hydrogel became turbid at body temperature in comparison to the initial sol (Figure 3).

#### 2.2.3. Rheology of Hydrogels

Rheological analysis can provide information regarding the ability of gel formulations to spread on the skin. The rheological properties of the gel can also affect the drug release kinetics and therefore, the study of these properties is essential. The viscosity of the prepared hydrogel remained higher between 15–36 °C, and then started to decline until 50 °C, showing the viscoelastic behavior of the hydrogels. Viscosity of nanoparticles incorporated hydrogels was higher than the simple hydrogels. This was due to the presence of polymer alginate in 5FU-Alg-Np-HG. The hydrogel becomes more viscous at body temperature as compared to the room temperature. Similar results were also previously reported by [40]. Chitosan a positive charge polymer interacts electrostatically with gelatin and form hydrogels. Gelatin temperature of hydrogels is determined by several factors including GP content, chitosan concentration, polymers molecular weight and its degree of deacetylation [41]. The elastic modulus (*G*′) of the prepared hydrogel increase gradually after 30 °C, which confirms the thermosensitive behavior of the prepared hydrogels. On the other hand, the viscous modulus (*G*″) remained constant [42]. Higher values of *G*′ indicates mechanical reinforcement of the gel under body temperature (37 °C) conditions, showing a stronger hydrogel network [42].

**Table 2:** Physicochemical properties of the nanoemulsion.

F. Code	pH		Viscosity (Pa)	
	25 °C	37 °C	25 °C	37 °C
5FU-HG	5.96 ± 1.63	6.53 ± 1.28	57.5 ± 2.37	66.16 ± 2.28
5FU-Alg-Np-HG	6.27 ± 1.92	6.83 ± 1.91	63.6 ± 2.91 *	78.32 ± 2.95 *

Note: * *p* < 0.05.

#### 2.2.4. Surface Morphology

**Figure 4:**
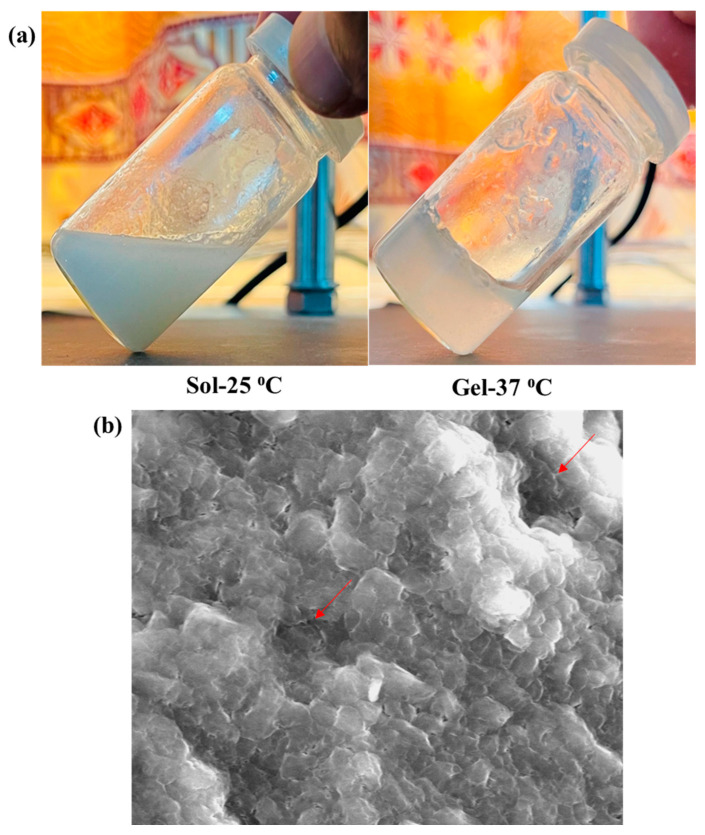
(**a**) Sol-Gel transition and (**b**) SEM of 5FU-Alg-Np-HG.

The surface morphology of the prepared hydrogel (5FU-Alb-Np-HG) was investigated using scanning electron microscopy. It was observed that the prepared hydrogels exhibited a dense and porous network structure as shown by red arrows in SEM image (Figure 4). The pores in the hydrogel are due to the existence of nanoparticles in the fabric. The presence of pores helps in the release of drug/nanoparticles from the polymer network. The dense structure shows the formation of more stable hydrogels.

#### 2.2.5. Swelling Studies

**Figure 5:**
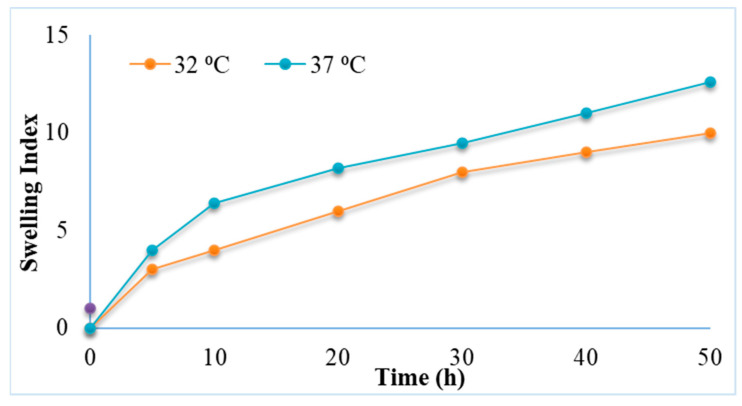
Swelling index of 5FU-Alg-Np-HG at 32 and 37 °C.

Swelling is described as a phenomenon in which water and/or biological fluids are retained in a polymer network. Swelling ability of the prepared hydrogels was evaluated by monitoring the mass changes during incubation in the dissolution medium. The swelling studies of chitosan-gelatin-based hydrogels containing 5FU-Alg nanoparticles at temperatures 32 and 37 °C and pH at 5.5 and 7.4 were performed. The capability of a hydrogel to swell depends on its chemical structure and the characteristics of its medium. It was found that the swelling of hydrogel increases with increase in temperature from 32 to 37 °C (Figure 5). This might be due to the hydrogen bond of the amine group in chitosan may dissociate with the water molecules inside the hydrogel network. As a result, the polymer chains in the hydrogel relax, which leads to increased swelling [43]. Swelling of hydrogels helps in control/sustain the release of 5FU from the hydrogel matrix. Higher swelling at 37 °C and pH 7.4 indicates more control over the release of 5FU in the deeper layers of skin.

### 2.3. In Vitro Release Studies

**Figure 6:**
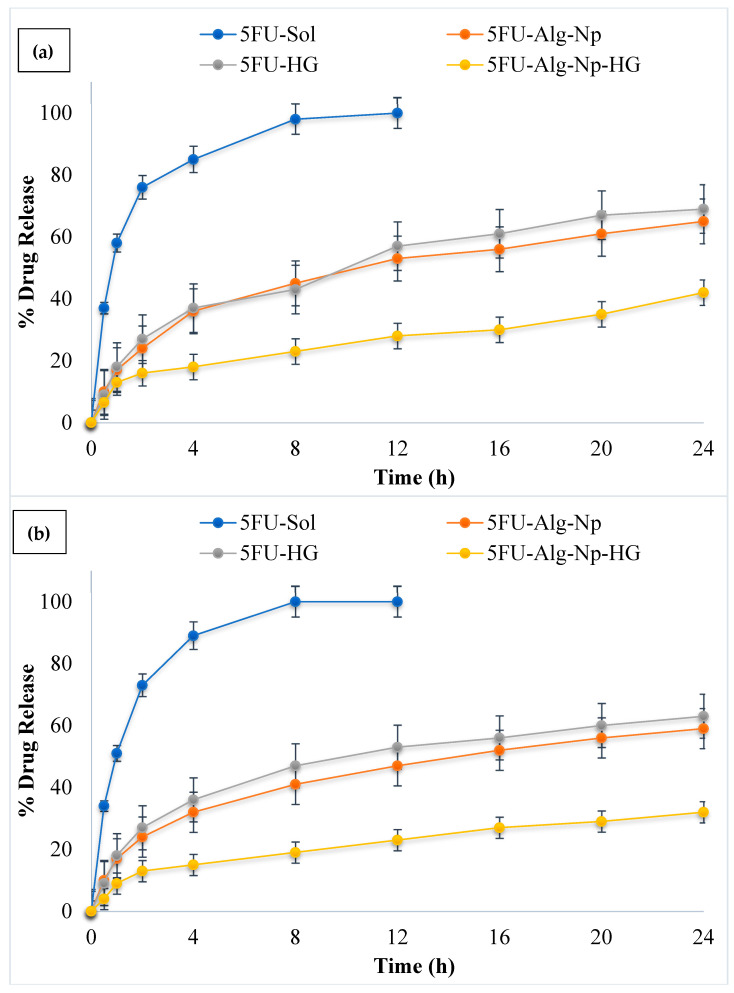
Release profile of 5FU at (**a**) 32 °C and pH 5.5; (**b**) 37 °C and pH 7.4.

The release of drugs from polymeric materials can be controlled by a number of physical processes, such as hydration, swelling, gel formation, drug diffusion from the matrix, and the erosion of the matrix [44]. The release profile of 5FU from the nanoparticles and hydrogel was investigated in phosphate buffer pH 5.5 (in-accordance with skin pH) and phosphate buffer pH 7.4 (in-accordance with blood pH) at a temperature 32 °C (in-accordance with skin temperature) and 37 °C (in-accordance with blood temperature), respectively (Figure 6a,b). The simple 5FU solution (in phosphate buffer pH 7.4) without a carrier was also explored as a reference. The results show that drug solution releases 5FU upto 97% in 6 h. Alginate nanoparticles significantly slowed down/control the release of 5FU as compared to solution (ANOVA; *p* < 0.05). In case of nanoparticles, it was found that rapid release of 5FU occurs during the first 120 min, followed by slower/sustained release. A much slower rate of release was observed in case of nanoparticles incorporated hydrogels with 45% release in 24 h. The initial burst release from the hydrogel might be due to weak absorption of the drug/nanoparticles in the hydrogel network. Furthermore, it was found that the release of 5FU was more sustained from the hydrogels at 37 °C as compared to 32 °C (ANOVA, *p* < 0.05). This was due to higher swelling of hydrogels at higher temperature (37 °C). The release of 5FU from solution and nanoparticles did not change significantly when the temperature increased from 32 to 37 °C.

Thus, the results indicate that the 5FU drug in the nanoparticles and hydrogels is highly protected from the physiological environment, and the release behavior from the nanoparticles and hydrogels is modulated by changes in environmental pH and temperature. It was noted that the drug release behavior from the chitosan-based hydrogels is similar to a previous study conducted by [42]. A Korsmeyer-Peppas model was used to determine the mechanism of drug release from the 5FU-Alg-Np-loaded chitosan-gelatin hydrogels. The n value found for the prepared hydrogel was 0.832, which indicates that the release of 5FU from the formulated system was due to diffusion, followed by swelling and erosion.

### 2.4. Ex Vivo Permeation and Retention Studies

**Figure 7:**
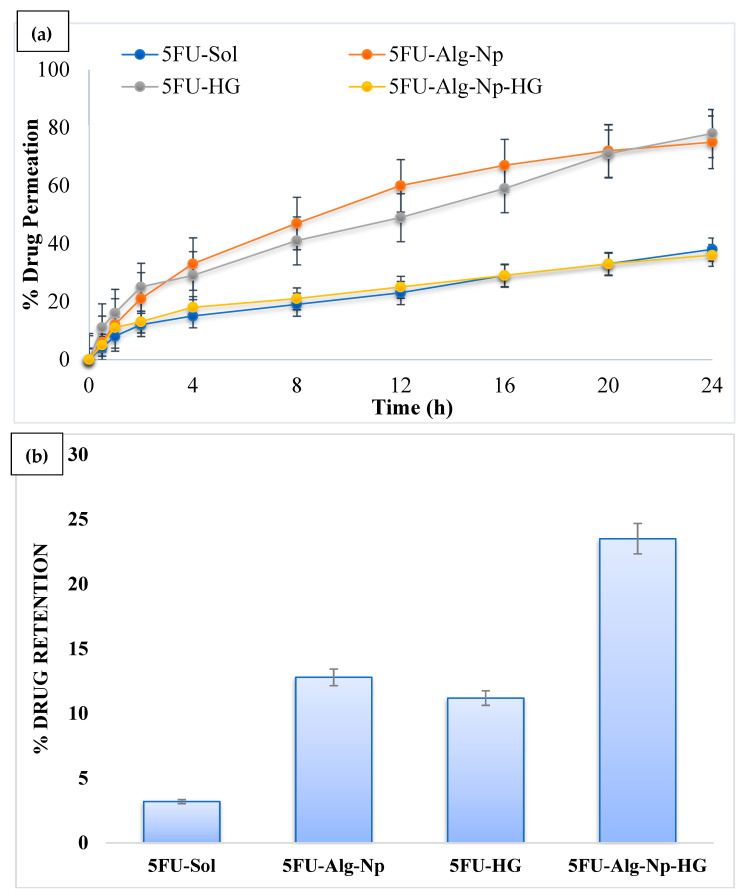
(**a**) Permeation profile and (**b**) skin-related drug retention of 5FU.

Ex vivo permeation studies were conducted for the formulations across the rat’s skin. The permeation of 5FU across the skin was higher in the case of nanoparticles as compared to hydrogels (ANOVA, *p* < 0.05) (Figure 7a). This was due to the smaller size and negative charge of the nanoparticles. However, when comparing nanoparticles vs. hydrogels, it was found that the permeability of 5FU was weaker when incorporated in gel. This was due to the additional processes of diffusion of 5FU from the gel base. The permeability of simple hydrogel was also higher than the 5FU-Alg-Np-HG. This was due to the lower release of 5FU from 5FU-Alg-Np-HG. Skin has the complex structure and acts as the main barrier against the entry of foreign chemicals. The stratum corneum, the outermost layer of skin, has highly organized structure and is main barrier and hence hinders drug penetration. The polymers, i.e., alginate and chitosan, are the main components of nanoparticles and hydrogels, play a role in determining drug permeation. Alginate and chitosan both interact with skin components proteins and lipids and helps in drug permeation. Chitosan as a matrix-forming polymer was also previously used for topical hydrogel formulations due to its permeation enhancer properties. Chitosan reversibly fluidizes the skin lipids and proteins, which results in the permeation of 5FU [45].

On the other hand, skin-related drug retention of 5FU-Alg-Np-HG was significantly higher than the nanoparticles and simple hydrogels (ANOVA, *p* < 0.05) (Figure 7b). This was due to higher swelling of 5FU-Alg-Np-HG at body temperature and pH (37 °C and 7.4). Higher skin-related drug retention and lower permeation is required to target the local skin cancers. The 5FU-Alg-Np-HG solution is an ideal delivery system with higher skin-related drug retention and lower permeation for the treatment of local skin cancer.

### 2.5. In Vivo Studies

**Figure 8:**
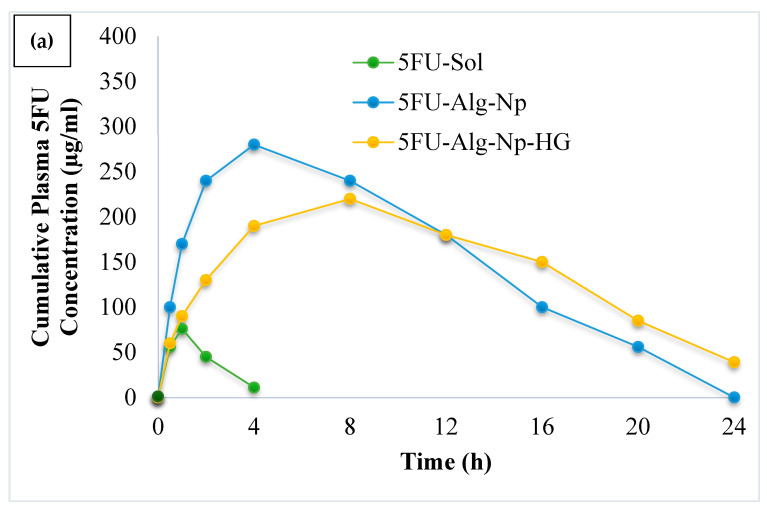

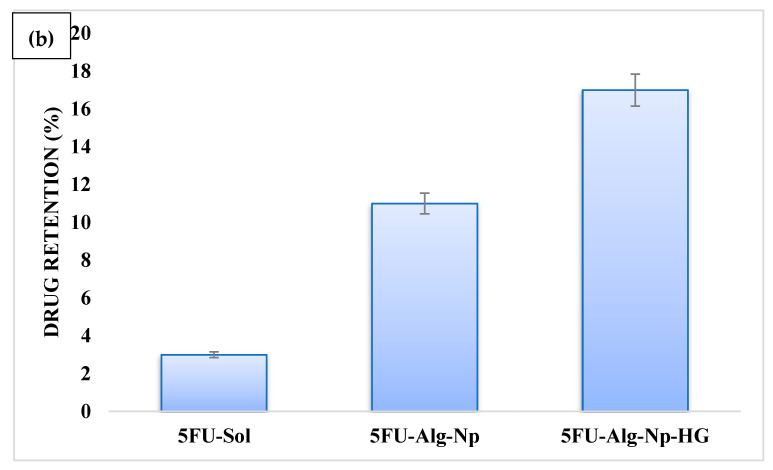
(**a**) Plasma drug profile and (**b**) skin-related drug retention of 5FU.

The mean plasma drug concentration vs. time was plotted and is shown in Figure 8. It was observed that hydrogels (5FU-Alg-Np-HG) controlled the release as effectively as pharmacokinetic parameters when compared to nanoparticles (5FU-Alg-Np) and solution (5FU sol). Short t_max_ and C_max_ values were observed for 5FU sol, this was due to inability of solution to penetrate the skin. The C_max_ was slightly higher for 5FU-Alg-Np as compared to hydrogels. This was attributed to the smaller size, higher drug release and ex vivo permeation of nanoparticles. The half-life and AUC were found higher in case of 5FU-Alg-Np-HG as compared to other formulations. The longer half-life shows prolonged delivery of 5FU from the hydrogel in comparison to solution and nanoparticles. The t_1/2_ of the drug is an important parameter and it controls the degree of drug accumulation, concentration fluctuations and the time taken to reach equilibrium [32]. Findings from our study reflect an important indication on the in-vivo sustained performance of hydrogels (5FU-Alg-Np-HG) after single application. Hence, the frequency of the dosage regimen, patient compliance and continuation of treatment for long time could be enhanced markedly by using 5FU-Alg-Np-HG. These findings are in correlation with the in vitro and ex vivo studies. Skin-related drug retention was significantly higher when 5FU-Alg-Np-HG was applied on the rats skin (ANOVA, *p* < 0.05). Higher skin-related drug retention is helpful in treating local skin cancers.

## 3. Conclusions

In this study, 5FU alginate nanoparticles were prepared using spray drying technique. The prepared nanoparticles were 202–254 nm in size, had a zeta potential of −43 to −38 mV, drug content of 78%, and exhibited aspherical shape. The nanoparticles were incorporated into chitosan-gelatin hydrogels to prevent the premature release of 5FU at the surface of skin. The release of 5FU from nanoparticles was higher as compared to the hydrogels. This was attributed to the additional processes of diffusion of 5FU from the gel base. Furthermore, 5FU was released from the hydrogels using diffusion mechanism followed by swelling and erosion. The skin-related drug retention was significantly higher in case of hydrogels as compared to nanoparticles. The reason behind this is the higher swelling of hydrogels in lower skin layers, which helps in the prolonged retention of 5FU in the skin. Similar results for hydrogels were also observed for in vivo studies. Chitosan-gelatin hydrogels containing 5FU-alginate nanoparticles is an ideal delivery system for the treatment of localized skin cancer.In future, the developed hydrogel system can be evaluated for its efficacy against skin cancers, such as melanoma, using nude mice.

## 4. Materials and Methods

### 4.1. Materials

The drug of interest, 5-flourouracil, wasobtained from Sigma-Aldrich (lot# A263299) (Sigma-Aldrich, Inc. St. Louis, MO, USA). Alginate (mol wt, 150–190 kDa; Sigma-Aldrich, Inc. St. Louis, MO, USA) was used for nanoparticle preparation. Chitosan (degree of deacetylation, 83%; mol wt, 310–375 kDa) and gelatin (mol wt, 300–350 kDa; G2500, Sigma, Inc. St. Louis, MO, USA) were used in hydrogel preparation. Acetic acid and methanol were obtained from Merck (Merck KGaA, Darmstadt, Germany). All the chemicals used were of analytical grade.

### 4.2. Preparation of Nanoparticles

The 5FU nanoparticles were prepared using a nano spray dryer (Pilotec YC-015, Shangai, China). Alginate was used as a model polymer and dissolved in distilled water (1% *w*/*w*) under continuous stirring at 1000 rpm. In addition, 0.3% *w*/*w* tween 80 was also added to the alginate solution as a surfactant. The 5FU (0.5% *w*/*w*) was taken and dissolved in distilled water, and the 5FU solution was then transferred to the alginate solution under constant stirring at 1000 rpm for 2 h. The drug polymer solution was then subjected to the spray dryer for nanoparticle preparation. The process parameters used were same as previously reported by our laboratory [46]. The inlet temperature was 70 °C, the outlet temperature was 40 °C, and the drug polymer solution was fed at the rate of 10 mL/min. Pressure was maintained at 6 bar, and actuator vibration frequency was kept at 60 kHz. The sample was collected from the collecting chamber with the help of rubber spatula and stored in a desiccator.

### 4.3. Characterization of Nanoparticles

#### 4.3.1. Size, Zeta Potential, and Surface Morphology

A DLS (dynamic light scattering) zetasizer (Malvern Zetasizer, Malvern Instruments Ltd., Worcestershire, UK) was used to measure the size and zetapotential of the prepared 5FU-Alg nanoparticles. The nanoparticles were taken and suspended/dispersed in 2 mL of ethanol (0.2% *w*/*v*) and determined on zetasizer in triplicate; the results were averaged.

SEM (scanning electron microscope) (Zeiss supra 40 VP scanning electron microscope) was used to investigate the surface morphology of the prepared 5FU-Alg nanoparticles and hydrogels. The prepared nanoparticles were taken and mounted on aluminum stubs with the help of double-sided conducting carbon tapes. The stubs were then coated with a 50/50 mixture of Pt/Pdin order to minimize the surface charging. Finally, the samples were scanned at an accelerating voltage of 10 kV, and photographs with different magnifications were taken.

#### 4.3.2. Percent Drug Content

A subsample of 15 mg of 5FU-Alg-Np was taken and dissolved in phosphate buffer pH 7.4 (30 mL) under continuous stirring at 1000 rpm for time period of 2 h. The sample was then taken, filtered using a syringe filter (0.45 µm), and then analyzed on a UV-Visible spectrophotometer (Shimadzu 1800, Nakagyo-Ku, Kyoto, Japan) at wavelength 265 nm [46]. The percent drug content was calculated using Equation (1):

(1) $$\%DC=\frac{Amount\;of\;Drug\;in\;Np}{Amount\;of\;Drug\;in\;Formulation}\times 100$$

### 4.4. Preparation of Chitosan-Gelatin Hydrogels Containing 5FU-Alginate Nanoparticles

Chitosan-gelatin hydrogels were prepared using previously developed method reported by Cheng et al., 2018 [47], with slight modifications. Briefly, chitosan (1 g) and gelatin (0.25 g) solutions were prepared by dissolving both in 50 mL of 1% *v*/*v* acetic acid solution. Glycerol 2-phosphate disodium salt hydrate (44.4% *w*/*v*) (GP) (G5422, Sigma, Inc. St. Louis, MO, USA) solution was then added dropwise to the chitosan-gelatin solution under continuous stirring in an ice-water bath. The pH value of chitosan-gelatin solution was adjusted to 7.4 by adding GP solution. The chitosan-gelatin-GP solution was stored at 4 °C and utilized as a controlled release system for both 5FU and 5FUnanoparticles. Subsequently, 5FU and 5FU-NPs were added to the chitosan-gelatin-GP solution under stirring and cooled in an ice-water bath for 30 min. The hydrogel was placed in an ultrasonic bath for further 30 min to eradicate entrapped air bubbles. The developed hydrogels were stored at 4 °C until further use [47].

### 4.5. Characterization of Hydrogels

#### 4.5.1. ATR-FTIR Analysis

The ATR-FTIR analysis of 5FU, alginate, chitosan, 5FU-Alg nanoparticles and hydrogel were performed using PerkinElmer ATR-FTIR spectrometer (L1600300; PerkinElmer, Beaconsfield, UK). The sample was placed on zinc selenide, and the spectra was recorded in range of 600–4000 cm^−1^ (32 scans per minute with a resolution of 4 cm^−1^) in transmission mode. Three spectra of each sample were taken and the results were averaged.

#### 4.5.2. pH and Visual Transparency of Hydrogels

A digital pH meter (Mettler-Toledo GmbH, Gieben, Germany) was used to measure the pH of the chitosan-gelatin hydrogel at a temperature of 25 °C. All measurements were performed in a triplicate and results averaged.

Transparency of the chitosan-gelatin hydrogel was determined at different temperatures (25–37 °C). A transparent solution indicates that all the added ingredients are homogeneously mixed [42].

#### 4.5.3. Gelation Time

The vial inversion method was used to measure the gelation time of the chitosan-gelatin hydrogel. The hydrogel components were mixed in a glass sample vial and incubated at 37 °C using water bath immediately after mixing and inverted every minute. The gelation time refers to the time required for “no flow” to be observed for hydrogel formulation [41].

#### 4.5.4. Rheological Measurements

The rheological properties of the prepared hydrogels (chitosan-gelatin-based hydrogel containing 5FU and 5FU-Alg-Np) were analyzed using a rheometer (Viscotester IQ AIR, HAAKE, Germany) (with a 60-mm diameter plate geometry with a gap of 1 mm). The sample of prepared hydrogel was added to the plate of rheometer at a temperature of 25 °C. The elastic modulus (*G′*) and viscous modulus (*G″*) were analyzed at a gap of 1 mm and a fixed frequency of 1.0 Hz. Gelation temperature of the samples was analyzed in a range from 25 °C to 40 °C at a rate of 0.5 °C per minute. The sol-gel transition time of the samples was analyzed at 34 °C.

#### 4.5.5. Swelling Studies

The hydrogels were freeze dried using freeze dryer (Freezone floor top freeze dryer, Labconco, Kansas City, MO, USA) at −50 °C for 24 h. The freeze-dried hydrogels were immersed in phosphate buffer saline (pH 7.4) at 37°C with shaking at 100 rpm. At predetermined time intervals, the hydrogels were removed from the PBS, the liquid was removed from the surface of hydrogel with tissue paper, and then the hydrogel was weighed [48].

The swelling index was calculated using Equation (2);

SI = (St − Si)/Si ×100%(2)

where Si = initial dry hydrogel weight, and St = wet hydrogel weight at time t.

### 4.6. In Vitro Release Studies

In vitro release studies were performed across synthetic membrane (Tyfran membrane; pore size 0.45 µm) using Franz diffusion cell. The donor compartment of the Franz diffusion cell was filled with drug polymer solution-nanoparticle-hydrogel and receptor compartment with release medium (phosphate buffer pH 5.5 and 7.4). The temperature of the medium was maintained at 32 and 37 °C. At specific time intervals (0, 0.5, 1, 2, 4, 8, 12, 16, 20, and 24 h), 2 mL of sample was collected and analyzed on UV spectroscopy at 265 nm [38]. The in vitro release data obtained, were fitted into Korsmeyer-Peppas equation (Equation (3)) for investigation of drug release mechanism.

Mt/M∞ = kt^n^ (3)

### 4.7. Ex Vivo Permeation and Skin-Related Drug Retention Studies

The ex vivo permeation study of prepared nanoparticles and hydrogels was performed on Franz diffusion cell using the rats’ skin as a membrane. The experiment was performed using the following working conditions: (i) receptor medium, phosphate buffer saline at pH 7.4; (ii) temperature, 37 °C ± 2 °C, (iii) rotation, 100 rpm. At predetermined time intervals, 2 mL of receiving solution was taken and replaced with fresh medium to maintain the sink condition. UV spectroscopy at 265 nm was used to determine the concentration of drug in the receiver medium.

After the permeation experiment, skin was removed from the Franz diffusion cell and surface was cleaned with phosphate buffer saline. The skin (exposed area to permeation experiment) was cut into small pieces with scissor, and transfer to beaker containing 20 mL of PBS pH 7.4. Subsequently, 5 mL of methanol was added for homogenization of skin and to extract the 5FU from the tissues. The solution was centrifuged for 5 min at 5000 rpm. The supernatant was taken and drug was quantified by means of a UV spectrophotometer at 265 nm [49].

### 4.8. In Vivo Studies

Sprague Dawley rats were used for in vivo studies. The animal procedures were approved by the Ethical Review Board, Gomal University Pakistan. For in-vivo tests of 5FU-Alg-Np and Hydrogels,15 male Sprague Dawley rats, weighing 200–250 g, were used. The rats were divided into three groups (GA=5FU sol, GB=5FU-Alg-Np, and GC=5FU-Alg-Np-HG), each group contains five rats (n = 5). The rats were anesthetized using ketamine/xylazine mixture, and then the dorsal region was shaved with sharp blades. After the application of formulations, 0.5 mL of blood sample was collected at specific intervals (0, 0.5, 2, 4, 8, 16 and 24) from the retro-orbital vein. The plasma drug concentrations were quantified using high-performance liquid chromatography (HPLC) and our previously developed method [49]. Pharmacokinetic parameters were calculated using Kinetica 5.0 software.

### 4.9. Statistical Analysis

All the experiments were performed in triplicate, and the results are presented as the mean values ± standard error (SE). The statistical analysis was performed using Student’s *t*-test and ANOVA, followed by post-hoc tests using Tukey’s honestly significant difference test, when required. The differences are considered significant at *p* < 0.05.

## Figures and Tables

**Table 1:** Size, zeta potential, and %DC of nanoparticles.

F. Code	Size (nm)	Zeta Potential	PDI	%DC
Alg-Np	202.35 ± 3.57	−43.67 ± 2.58	0.33 ± 0.27	-
5FU-Alg-Np	254.71 ± 3.98 *	−38.72 ± 2.91	0.38 ± 0.24	78.23 ± 3.26

Note: * *p* < 0.05.

## Data Availability

Not applicable.

## References

[1] Correa S., Grosskopf A.K., Lopez Hernandez H., Chan D., Yu A.C., Stapleton L.M., Appel E.A. (2021). Translational Applications of Hydrogels. Chem. Rev..

[2] Cascone S., Lamberti G. (2020). Hydrogel-based commercial products for biomedical applications: A review. Int. J. Pharm..

[3] Mahmood A., Patel D., Hickson B., Desrochers J., Hu X. (2022). Recent Progress in Biopolymer-Based Hydrogel Materials for Biomedical Applications. Int. J. Mol. Sci..

[4] Pita-López M.L., Fletes-Vargas G., Espinosa-Andrews H., Rodríguez-Rodríguez R. (2021). Physically cross-linked chitosan-based hydrogels for tissue engineering applications: A state-of-the-art review. Eur. Polym. J..

[5] Zhou G., Groth T. (2018). Host Responses to Biomaterials and Anti-Inflammatory Design—A Brief Review. Macromol. Biosci..

[6] Carrillo-Castillo T.D., Luna-Velasco A., Zaragoza-Contreras E.A., Castro-Carmona J.S. (2021). Thermosensitive hydrogel for in situ-controlled methotrexate delivery. E-Polymers.

[7] Dreiss C.A. (2020). Hydrogel design strategies for drug delivery. Curr. Opin. Colloid Interface Sci..

[8] Kocen R., Gasik M., Gantar A., Novak S. (2017). Viscoelastic behaviour of hydrogel-based composites for tissue engineering under mechanical load. Biomed. Mater..

[9] Bordbar-Khiabani A., Gasik M. (2022). Smart Hydrogels for Advanced Drug Delivery Systems. Int. J. Mol. Sci..

[10] Caló E., Khutoryanskiy V.V. (2015). Biomedical applications of hydrogels: A review of patents and commercial products. Eur. Polym. J..

[11] Marques A.C., Costa P.J., Velho S., Amaral M.H. (2021). Stimuli-responsive hydrogels for intratumoral drug delivery. Drug Discov. Today.

[12] Lv H., Li L., Sun M., Zhang Y., Chen L., Rong Y., Li Y. (2015). Mechanism of regulation of stem cell differentiation by matrix stiffness. Stem Cell Res. Ther..

[13] Montoya C., Du Y., Gianforcaro A.L., Orrego S., Yang M., Lelkes P.I. (2021). On the road to smart biomaterials for bone research: Definitions, concepts, advances, and outlook. Bone Res..

[14] Tang G., Tan Z., Zeng W., Wang X., Shi C., Liu Y., He H., Chen R., Ye X. (2020). Recent Advances of Chitosan-Based Injectable Hydrogels for Bone and Dental Tissue Regeneration. Front. Bioeng. Biotechnol..

[15] Ul-Islam S., Butola B.S. (2019). Advanced Functional Textiles and Polymers: Fabrication, Processing and Applications.

[16] Askari E., Seyfoori A., Amereh M., Gharaie S.S., Ghazali H.S., Ghazali Z.S., Khunjush B., Akbari M. (2020). Stimuli-responsive hydrogels for local post-surgical drug delivery. Gels.

[17] Chakraborty D.D., Nath L.K., Chakraborty P. (2018). Recent Progress in Smart Polymers: Behavior, Mechanistic Understanding and Application. Polym. Technol. Eng..

[18] Erukhimovich I., de la Cruz M.O. (2004). Phase equilibria and charge fractionation in polydisperse polyelectrolyte solutions. arXiv.

[19] Kopač T., Krajnc M., Ručigaj A. (2021). A mathematical model for pH-responsive ionically crosslinked TEMPO nanocellulose hydrogel design in drug delivery systems. Int. J. Biol. Macromol..

[20] Yin Z.C., Wang Y.L., Wang K. (2018). A pH-responsive composite hydrogel beads based on agar and alginate for oral drug delivery. J. Drug Deliv. Sci. Technol..

[21] Nazir S., Umar Aslam Khan M., Shamsan Al-Arjan W., Izwan Abd Razak S., Javed A., Rafiq Abdul Kadir M. (2021). Nanocomposite hydrogels for melanoma skin cancer care and treatment: In-vitro drug delivery, drug release kinetics and anti-cancer activities. Arab. J. Chem..

[22] Tian B., Liu Y., Liu J. (2021). Smart stimuli-responsive drug delivery systems based on cyclodextrin: A review. Carbohydr. Polym..

[23] Nguyen C.H., Banh K.S., Dang C.H., Nguyen C.H., Nguyen T.D. (2022). Β-Cyclodextrin/Alginate Nanoparticles Encapsulated 5-Fluorouracil As an Effective and Safe Anticancer Drug Delivery System. Arab. J. Chem..

[24] Ma J., Wang Y., Lu R. (2022). Mechanism and Application of Chitosan and Its Derivatives in Promoting Permeation in Transdermal Drug Delivery Systems: A Review. Pharmaceuticals.

[25] Lukin I., Erezuma I., Maeso L., Zarate J., Desimone M.F., Al-Tel T.H., Dolatshahi-Pirouz A., Orive G. (2022). Progress in Gelatin as Biomaterial for Tissue Engineering. Pharmaceutics.

[26] Xing Q., Yates K., Vogt C., Qian Z., Frost M.C., Zhao F. (2014). Increasing mechanical strength of gelatin hydrogels by divalent metal ion removal. Sci. Rep..

[27] Zhang D.Y., Shen X.Z., Wang J.Y., Dong L., Zheng Y.L., Wu L.L. (2008). Preparation of chitosan-polyaspartic acid-5-fluorouracil nanoparticles and its anti-carcinoma effect on tumor growth in nude mice. World J. Gastroenterol..

[28] Rata D.M., Cadinoiu A.N., Atanase L.I., Popa M., Mihai C.T., Solcan C., Ochiuz L., Vochita G. (2021). Topical formulations containing aptamer-functionalized nanocapsules loaded with 5-fluorouracil—An innovative concept for the skin cancer therapy. Mater. Sci. Eng. C.

[29] Benson H.A.E., Grice J.E., Mohammed Y., Namjoshi S., Roberts M.S. (2019). Topical and Transdermal Drug Delivery: From Simple Potions to Smart Technologies. Curr. Drug Deliv..

[30] Alkilani A.Z., McCrudden M.T.C., Donnelly R.F. (2015). Transdermal drug delivery: Innovative pharmaceutical developments based on disruption of the barrier properties of the stratum corneum. Pharmaceutics.

[31] Sarfaraj Hussain M., Azam F., Ahmed Eldarrat H., Haque A., Khalid M., Zaheen Hassan M., Ali M., Arif M., Ahmad I., Zaman G. (2022). Structural, functional, molecular, and biological evaluation of novel triterpenoids isolated from Helichrysum stoechas (L.) Moench. Collected from Mediterranean Sea bank: Misurata–Libya. Arab. J. Chem..

[32] Honary S., Zahir F. (2013). Effect of zeta potential on the properties of nano-drug delivery systems—A review (Part 1). Trop. J. Pharm. Res..

[33] Tang L., Yang X., Yin Q., Cai K., Wang H., Chaudhury I., Yao C., Zhou Q., Kwon M., Hartman J.A. (2014). Investigating the optimal size of anticancer nanomedicine. Proc. Natl. Acad. Sci. USA.

[34] Ghasemiyeh P., Mohammadi-Samani S. (2020). Potential of nanoparticles as permeation enhancers and targeted delivery options for skin: Advantages and disadvantages. Drug Des. Devel. Ther..

[35] Rodríguez-Rodríguez R., Espinosa-Andrews H., Morales-Hernández N., Lobato-Calleros C., Vernon-Carter E.J. (2019). Mesquite gum/chitosan insoluble complexes: Relationship between the water state and viscoelastic properties. J. Dispers. Sci. Technol..

[36] Raval N., Maheshwari R., Kalyane D., Youngren-Ortiz S.R., Chougule M.B., Tekade R.K. (2018). Importance of Physicochemical Characterization of Nanoparticles in Pharmaceutical Product Development.

[37] Darban Z., Shahabuddin S., Gaur R., Ahmad I., Sridewi N. (2022). Hydrogel-Based Adsorbent Material for the Effective Removal of Heavy Metals from Wastewater: A Comprehensive Review. Gels.

[38] Nawaz A., Latif M.S., Alnuwaiser M.A., Ullah S., Iqbal M., Alfatama M., Lim V. (2022). Synthesis and Characterization of Chitosan-Decorated Nanoemulsion Gel of 5-Fluorouracil for Topical Delivery. Gels.

[39] Lukić M., Pantelić I., Savić S.D. (2021). Towards optimal ph of the skin and topical formulations: From the current state of the art to tailored products. Cosmetics.

[40] Boisgard A.S., Lamrayah M., Dzikowski M., Salmon D., Kirilov P., Primard C., Pirot F., Fromy B., Verrier B. (2017). Innovative drug vehicle for local treatment of inflammatory skin diseases: Ex vivo and in vivo screening of five topical formulations containing poly(lactic acid) (PLA) nanoparticles. Eur. J. Pharm. Biopharm..

[41] Ohmes J., Saure L.M., Schütt F., Trenkel M., Seekamp A., Scherließ R., Adelung R., Fuchs S. (2022). Injectable Thermosensitive Chitosan-Collagen Hydrogel as A Delivery System for Marine Polysaccharide Fucoidan. Mar. Drugs.

[42] Ahsan A., Farooq M.A., Parveen A. (2020). Thermosensitive chitosan-based injectable hydrogel as an efficient anticancer drug carrier. ACS Omega.

[43] Singh A., Narvi S.S., Dutta P.K., Pandey N.D. (2006). External stimuli response on a novel chitosan hydrogel crosslinked with formaldehyde. Bull. Mater. Sci..

[44] Oh G.W., Nam S.Y., Heo S.J., Kang D.H., Jung W.K. (2020). Characterization of ionic cross-linked composite foams with different blend ratios of alginate/pectin on the synergistic effects for wound dressing application. Int. J. Biol. Macromol..

[45] Nawaz A., Wong T.W. (2018). Chitosan-Carboxymethyl-5-Fluorouracil-Folate Conjugate Particles: Microwave Modulated Uptake by Skin and Melanoma Cells. J. Investative Dermatol..

[46] Shah M.K.A., Azad A.K., Nawaz A., Ullah S., Latif M.S., Rahman H., Alsharif K.F., Alzahrani K.J., El-Kott A.F., Albrakati A. (2022). Formulation development, characterization and antifungal evaluation of chitosan nps for topical delivery of voriconazole in vitro and ex vivo. Polymers.

[47] Cheng Y.H., Ko Y.C., Chang Y.F., Huang S.H., Liu C.J.L. (2019). Thermosensitive chitosan-gelatin-based hydrogel containing curcumin-loaded nanoparticles and latanoprost as a dual-drug delivery system for glaucoma treatment. Exp. Eye Res..

[48] Tao J., Zhang Y., Shen A., Yang Y., Diao L., Wang L., Cai D., Hu Y. (2020). Injectable chitosan-based thermosensitive hydrogel/nanoparticle-loaded system for local delivery of vancomycin in the treatment of osteomyelitis. Int. J. Nanomed..

[49] Ullah S., Nawaz A., Farid A., Latif M.S., Fareed M., Ghazanfar S., Galanakis C.M., Alamri A.S., Alhomrani M., Mohammed S. (2022). Folate-Modified Chitosan 5-Flourouraci Nanoparticles-Embedded Calcium Alginate Beads for Colon Targeted Delivery. Pharmaceutics.

